# Dynamic responses in the human methylome to exertional heat exhaustion, heat injury, and heat stroke

**DOI:** 10.1186/s40246-026-01008-8

**Published:** 2026-06-19

**Authors:** Brandon M. Roberts, Ruoting Yang, Kari C. Goodwin, Aarti Gautam, Stacy Miller, Ida Nela Crespo Rosales, David W. DeGroot, Rasha Hammamieh, Nisha Charkoudian

**Affiliations:** 1 Experimental Therapeutics Branch, CIDR, Walter Reed Army Institute of Research, 503 Robert Grant Ave, Silver Spring, MD 20910 USA; 2 Integrative Systems Biology, CMPN, Walter Reed Army Institute of Research, Silver Spring, MD USA; 3 Oak Ridge Institute of Science and Education, Oak Ridge, TN USA; 4 Thermal and Medicine Mountain Division, Army Research Institute of Environmental Medicine, Natick, MA USA; 5 Culmen International, Alexandria, VA USA; 6 Army Heat Center, Ft Benning, GA USA

## Abstract

Exertional heat illness encompasses a continuum from heat exhaustion (EHE) to heat stroke (EHS), yet the molecular mechanisms remain poorly understood. DNA methylation offers a stable epigenetic signature linking environmental stress to gene regulation and long-term physiological outcomes. We profiled genome-wide DNA methylation in blood from active-duty service members hospitalized for EHE (*n* = 36), heat injury (EHI; *n* = 18), or EHS (*n* = 50). Blood was collected longitudinally and analyzed using the Illumina 850 K array, with normalization and time-course clustering (TCseq) to identify co-regulated CpG networks. Ingenuity Pathway Analysis was applied differentially methylated probes (*p* < 0.01, Δβ > 0.04). At diagnosis, both EHI and EHS shared hypomethylation in pathways regulating heat sensing, oxidative defense, and vascular tone. Over time, EHI exhibited adaptive methylation changes supporting neuronal repair, glucocorticoid regulation, and cytoskeletal stability. In contrast, EHS demonstrated sustained downregulation of metabolic and cardiovascular regulators, persistent inflammasome activation, and oxidative imbalance. Early patterns were shared between EHI and EHS, reflecting a common acute stress response, but later responses diverged: EHI showed partial epigenetic recovery, while EHS exhibited sustained metabolic suppression and inflammasome activation. These time-dependent methylation signatures identify potential molecular targets for promoting recovery and preventing long-term complications of heat illnesses.

**Supplementary Information:**

The online version contains supplementary material available at https://doi.org/10.1186/s40246-026-01008-8.

## Introduction

Exertional heat illness encompasses a clinical continuum including exertional heat exhaustion (EHE), exertional heat injury (EHI), and exertional heat stroke (EHS), all of which are characterized by hyperthermia associated with physical activity. EHI and EHS include varying degrees of organ dysfunction which can range from serious to fatal if not promptly treated [1, 2]. Operational settings place military personnel at particularly high risk, but substantial risk exists for athletes, first responders, agricultural and construction workers as well [3–6]. Thus, understanding exertional heat illnesses has broad public health implications, as prior heat illness and repeated heat exposure have been associated with increased long-term cardiovascular events and all-cause mortality [7–9].

DNA methylation has emerged as a mechanism underlying the biological adaptation to heat stress and recovery from heat illness [10, 11]. Methylation refers to the covalent attachment of a methyl group to the cytosine ring within cytosine-guanine (CpG sites) [12], and it may reflect prior environmental stress but may also be predictive of future gene expression regulations [13–15]. Environmental factors such as temperature, oxidative stress, and duration of exposure can change methylation patterns, leading to hypermethylation, which typically represses gene transcription by condensing the chromatin, or hypomethylation, which can allow access to DNA for transcription to occur, depending on the location they occur in DNA [16–18].

Preclinical models have provided some insight into how heat stress influences DNA methylation and downstream physiological responses. In cattle, exposure to heat stress induces hypomethylation in genes regulating hormone synthesis and secretion, including cortisol, aldosterone, and insulin pathways [13]. Studies in model organisms demonstrate that heat stress can activate innate immune signaling, enhance stress resistance, and extend lifespan, with epigenetic patterns transmitted across generations to confer survival benefits in offspring [9, 13]. Recent research has begun to reveal the role of epigenetic mechanisms in the response to heat stress in murine models [10, 19–21]. For instance, changes in DNA methylation patterns can regulate the expression of genes involved in the stress response, including cytokine signaling [22]. Similarly, EHS has been associated with post-recovery metabolic dysfunction, possibly linked to persistent epigenetic modifications affecting energy metabolism and mitochondrial regulation [23]. Together, these studies show that heat stress reshapes the epigenome and methylation may contribute to long-term thermotolerance or maladaptive remodeling.

To date, no epigenetic modifications have been characterized in humans immediately following collapse from an exertional heat illness. Understanding these epigenetic modifications is critical, as they may inform strategies (i.e., drug development) to mitigate the impact of heat-related injuries and influence recovery processes. Therefore, in the present study, we aimed to investigate the methylation patterns of DNA in individuals who collapsed from EHE, EHI, or EHS, starting with immediate post-collapse blood samples from the emergency department. Based on prior preclinical evidence that heat stress and EHS alter inflammatory, immune, metabolic, and organ-injury pathways, we hypothesized that loci related to inflammation and organ injury would show differential methylation across the clinical spectrum of exertional heat illness. Because no prior studies have characterized methylome-wide responses immediately following exertional heat illness in humans, this study was also designed as an exploratory analysis to identify severity- and recovery-associated epigenetic signatures.

## Methods

### Study approval

The study enrolled active-duty service members aged 17–45 who were brought to the Emergency Department and diagnosed with exertional EHE, EHI, or EHS. This research received approval from the U.S. Army Medical Research and Development Command Institutional Review Board (Protocol no. M-10872) and adhered to all provisions of the Declaration of Helsinki. Participants gave written informed consent either when they became medically stable during hospitalization or at their initial Heat Clinic follow-up visit if discharged. All participants signed HIPAA authorization forms.

### Study population

The study cohort consisted of 104 military personnel. Participants were categorized into three groups based on their clinical diagnosis: EHE (*n* = 36), EHS (*n* = 50), or EHI (*n* = 18). Peripheral blood samples were collected in PAXgene DNA tubes at an initial time point (T0) corresponding to the time of diagnosis and at follow-up intervals. Demographic and clinical data, including age, sex, Body Mass Index (BMI), Urine Specific Gravity (USG), Creatine Kinase, and Creatinine, were recorded. However, not all participants provided samples at every time point. As a result, sample sizes varied by group and time point. Analyses were conducted using an available-case approach, such that all subjects with valid measurements at a given time point were included in analyses for that time point. For example, at baseline (T0), samples were available from 39 EHS, 16 EHI, and 32 EHE participants.

### DNA extraction

Genomic DNA was extracted using the PAXgene Blood DNA Kit (Qiagen, Germantown MD, USA). Genomic DNA (500 ng) was treated with sodium bisulfite using the Zymo EZ96 DNA Methylation Kit (Zymo Research, Orange CA, USA), and genome-wide DNA methylation patterns were profiled using the Infinium HumanMethylation450 BeadChip (850 K) Kit (Illumina, Inc., San Diego CA, USA) [24, 25]. BeadChips were washed, and allele-specific single-base extension and staining with multiple layers of fluorescence was performed. BeadChip imaging was conducted using the Illumina iScan system (Illumina Inc., San Diego CA, USA). IDAT files containing the raw intensity signals were generated using Illumina’s iControl software.

### Quality control and normalization

All samples passed the log median intensity quality control of methylated and unmethylated channels assessed using the R minfi package v1.30.0. Three samples were filtered out as outliers identified by Principal Component Analysis. For each CpG site, minfi computes a β value, defined as the ratio between the fluorescence signal of the methylated allele (C) and the sum of the fluorescence signals of the methylated (C) and unmethylated (T) alleles; thus, higher β values correspond to higher methylation levels. Prior to normalization, probes with poor detection (average detection *P* value > 0.01), located on the X or Y chromosomes, mapping to multiple genomic locations, or overlapping known SNPs were removed using the ChAMP R package. β values were then normalized using the BMIQ method.

### Statistical and bioinformatic analysis

Statistical analyses were performed using R v 4.3.3, specifically with the packages Limma, TCseq, and tableone. Descriptive statistics were calculated for socio-demographic characteristics and clinical assessments. Continuous variables were summarized with means ± standard deviations (SD) and compared using one way ANOVA test, while categorical variables were summarized with frequencies and compared using χ2 tests. A paired t-test was used to compare methylation levels between different time points within the same individuals. We adopted a discovery threshold of *p* < 0.01 combined with Δβ > 0.04 to analyze differentially methylated loci via Ingenuity Pathway Analysis (v 01–08) to determine functional pathway enrichment, with enrichment defined by a p-value < 0.0001 and an absolute z-score > 0.5. For three-way comparisons, a pathway was considered significantly enriched if it met both of the following criteria: (i) at least one pairwise comparison yielded a *p*-value < 0.001, and (ii) the pathway demonstrated an absolute *z*-score greater than 0.5 across all comparisons.

### Statistical models

Differential methylation analyses were conducted using the Limma package with empirical Bayes moderation to identify significant differences associated with group status. We compared the DNA methylation profiles of participants with EHS and EHI to the EHE group at the initial timepoint and examined methylation patterns at follow-up timepoints to understand temporal changes. The DNA loci differentiating between groups at a given timepoint were modeled using a linear regression adjusted for age, sex, race, and blood cell-type composition, which is estimated from DNA methylation profiles (Houseman method). Specifically, the limma model can be summarized as:

$$ \begin{gathered} {\text{Methylation }}\sim {\text{ EHI}}\_{\mathrm{vs}}\_{\text{EHE }} + {\text{ Age }} \hfill \\ + {\text{ Sex }} + {\text{ Race }} + {\text{ Granulocyte }} + {\text{ Monocyte}} \hfill \\ \end{gathered} $$

$$ \begin{gathered} {\text{Methylation }}\sim {\text{ EHS}}\_{\mathrm{vs}}\_{\text{EHE }} + {\text{ Age }} \hfill \\ + {\text{ Sex }} + {\text{ Race }} + {\text{ Granulocyte }} + {\text{ Monocyte}} \hfill \\ \end{gathered} $$

### Time-course analysis

To analyze the dynamic changes in methylation over time, sample collection times were grouped into four distinct time bins: T1 (days 1–2), T2 (days 3–4), T3 (days 5–8), and T4 (> 8 days). To visualize the global, dynamic changes in methylation patterns, we employed Self-Organizing Maps, using the Kohonen package. This unsupervised neural network approach clusters the high-dimensional feature space of methylation data onto a two-dimensional grid, in this case, a 15 × 15 grid. In our implementation, we used 100 training iterations, which was sufficient for visual and quantitative stabilization of the cluster patterns. To identify groups of CpG sites with similar temporal patterns, a time-course fuzzy c-means clustering analysis was performed for the EHS and EHI groups using the TCseq R package. This method groups CpG probes into clusters based on their expression profiles over time, allowing for the identification of co-regulated networks. Normalized counts were transformed to log2 counts per million and scaled by gene. Clustering was carried out using the fuzzy c-means algorithm, and we evaluated candidate cluster numbers k = 2–8 by visual inspection of the cluster centroids and composition. We chose four clusters (k = 4) because this was the smallest number of clusters that clearly separated the major temporal patterns (sustained downregulation (SD), delayed downregulation (DD), delayed upregulation (DU), and transient upregulation (TU)), while additional clusters mostly split existing groups without adding new biological insight.

## Results

The demographic and clinical characteristics of the study cohort at baseline can be seen in Table 1. The distribution of heat-related illnesses into time bins can be found in Table 2.

**Table 1:** Demographic and clinical characteristics of the study cohort at the initial time point

Variable	Heat Exhaustion	Heat Injury	Heat Stroke	*p*-value
n	36	18	50	
Age (mean (SD))	22.00 (4.16)	24.89 (4.09)	24.44 (4.78)	0.022
Sex = M (%)	35 (97.2)	17 (94.4)	40 (80.0)	0.033
BMI (mean (SD))	26.43 (3.28)	25.98 (4.03)	27.42 (2.54)	0.158
USG (mean (SD))	1.02 (0.01)	1.02 (0.01)	1.01 (0.01)	0.031
Creatine Kinase (mean (SD))	953.74 (1068.91)	2107.76 (2265.07)	1506.94 (3435.12)	0.326
Creatinine (mean (SD))	1.22 (0.32)	1.75 (0.69)	1.72 (0.59)	< 0.001

Values are presented as mean (standard deviation) for continuous variables and n (%) for categorical variables. P-values for continuous variables were obtained using one-way ANOVA, and p-values for categorical variables were obtained using χ² tests

**Table 2:** Distribution of three heat-related illnesses in the study for samples

Time Bin	Time in Days (Mean ± SD)	Heat Exhaustion	Heat Stroke	Heat Injury
Only T = 1	—	28	9	5
T1 = (1, 2)	0.35 ± 0.48	14	45	18
T2 = (3, 4)	3.41 ± 0.50	3	37	6
T3 = (5, 6, 7, 8)	6.43 ± 1.19	0	52	6
T4 = (9–22)	11.91 ± 3.05	0	43	3

### Distinct epigenetic signatures at initial diagnosis (T0)

**Fig. 1:**
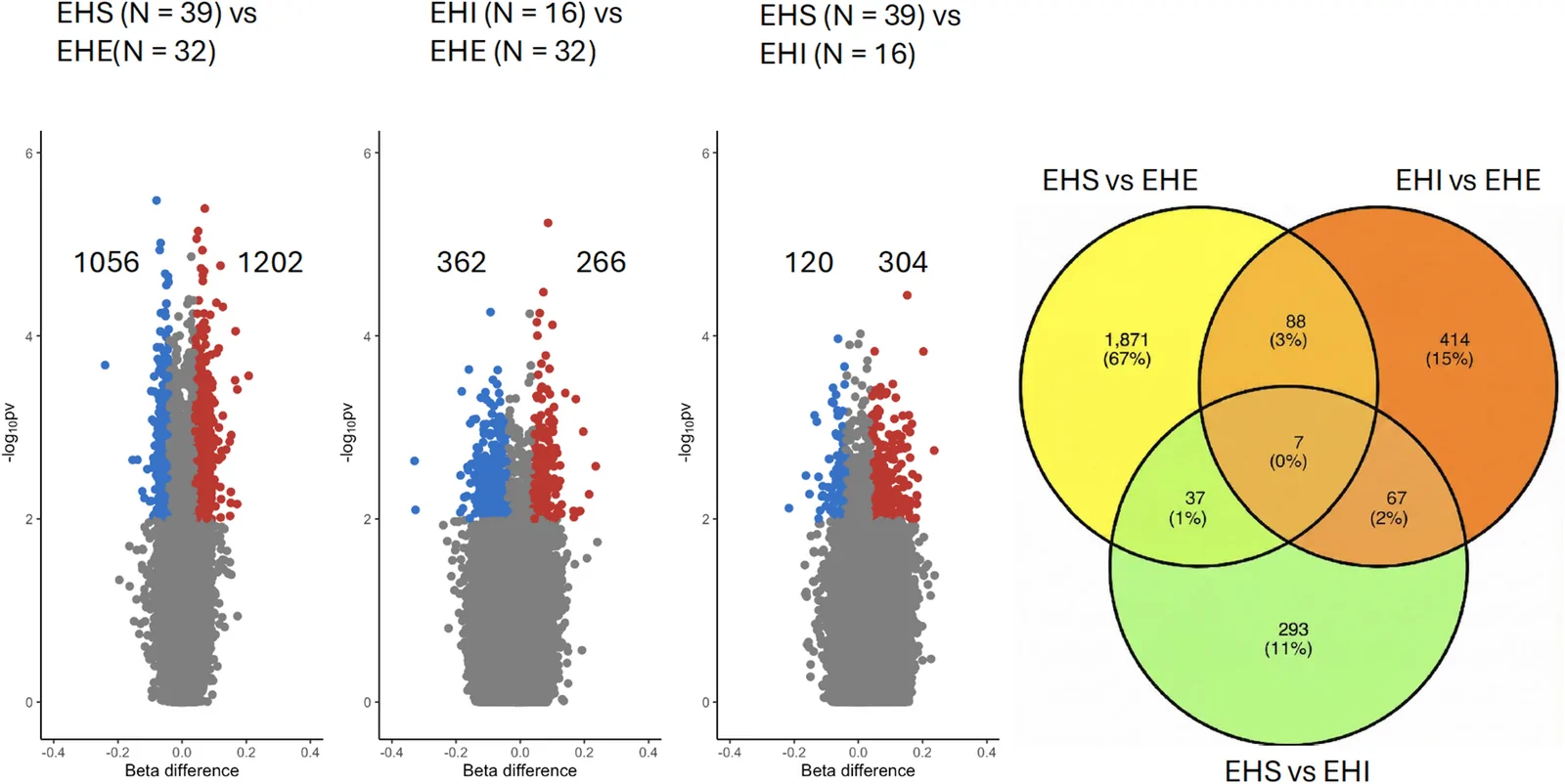
Differentially Regulated Methylation. Volcano plots illustrating differentially methylated probes (DMPs) at the initial time point (T0). **a** Compares Exertional Heat Stroke (EHS) to Exertional Heat Exhaustion (EHE), and the middle panel compares Exertional Heat Injury (EHI) to EHE, and the right panel compares EHS to EHI. Each point represents a CpG probe. Probes with significant p-values (*p* < 0.01) and absolute beta differences > 0.04 are highlighted in red and blue. **b** Venn diagram illustrating the overlap of differentially methylated probes identified in the EHS vs. EHE and EHI vs. EHE comparisons at T0

At the initial time point (T0), we identified differences in DNA methylation between the EHI and EHE group (Fig. 1A). The comparison between EHS (*n* = 39) and EHE (*n* = 32) revealed 2258 differentially methylated probes (DMPs). The comparison between EHI (*n* = 16) and EHE (*n* = 32) identified 628 DMPs, while EHS vs. EHI identified 424 DMPs. The genomic location of DMPs is available in the Supplementary Tables.

**Fig. 2:**
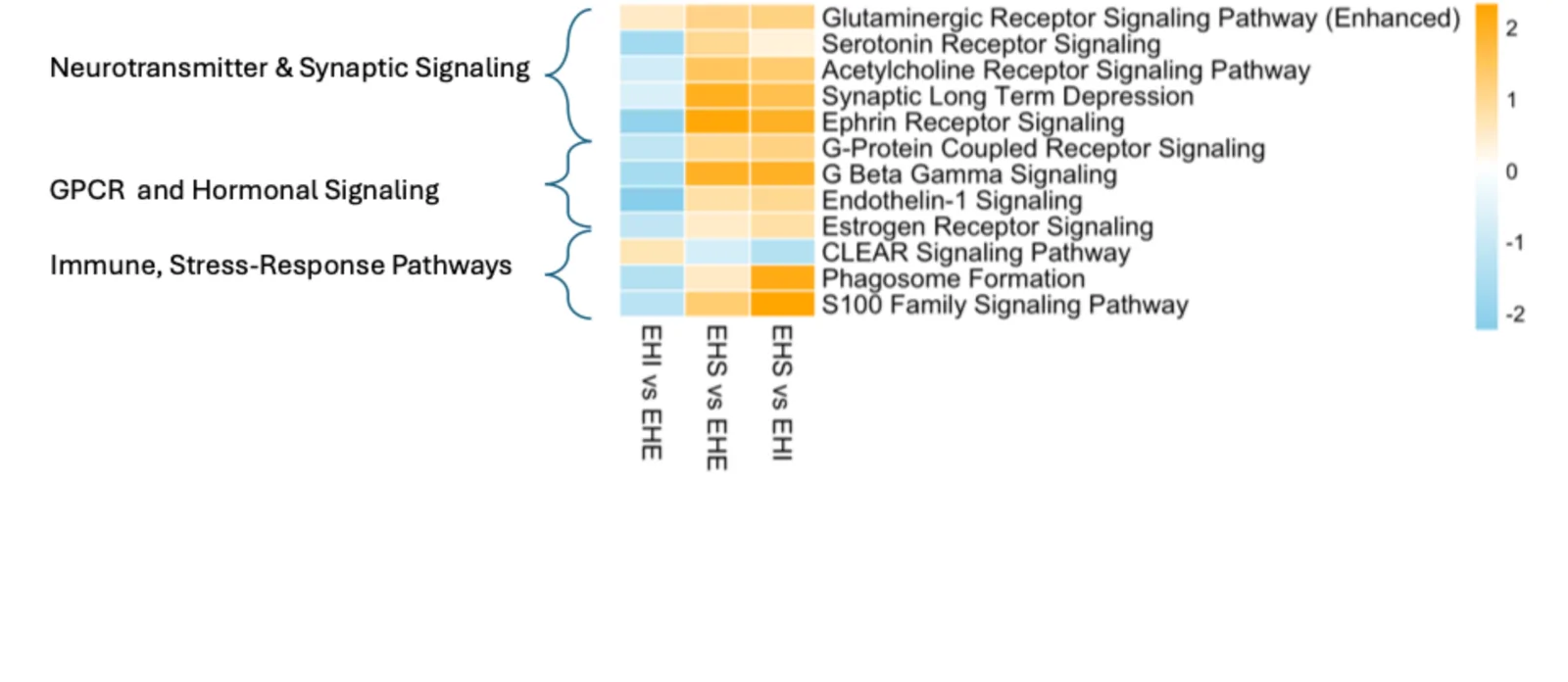
Heatmap of enriched canonical pathways for the Exertional Heat Stroke (EHS) vs. Exertional Heat Exhaustion (EHE) and Exertional Heat Injury (EHI) vs. EHE comparisons at T0. The color scale represents the z-score, with orange indicating predicted pathway activation and blue indicating predicted inhibition

To determine if EHS and EHI shared a common epigenetic signature, we compared the three lists of DMPs. Most loci were unique to the EHS vs. EHE contrast (1,871 loci; 67%), with smaller unique sets in EHI vs. EHE (414 loci; 15%) and EHS vs. EHI (293 loci; 11%). Only a limited number of loci were shared between two comparisons (EHS vs. EHE and EHI vs. EHE: 88 loci, 3%; EHS vs. EHE and EHS vs. EHI: 37 loci, 1%; EHI vs. EHE and EHS vs. EHI: 67 loci, 2%), and just 7 loci were common to all three contrasts, indicating largely distinct methylation signatures for each comparison (Fig. 1B). Figure S1 displays top 20 DMPs distinguishing EHS from HE, while Figure S2 displays top 20 DMPs distinguishing EHI from EHE. Pathway analysis of the DMPs unique to EHS and EHI revealed enrichment in several key biological domains (Fig. 2). For three-way comparisons, a pathway was considered significantly enriched if it met both of the following criteria: (i) at least one pairwise comparison yielded a p-value < 0.001, and (ii) the pathway demonstrated an absolute z-score greater than 0.5 across all comparisons.

### Longitudinal DNA methylation dynamics

T0 EHS–EHI DMPs were tracked across longitudinal response-pattern gene sets (Table 3). EHI exhibited greater persistence of T0 DMPs across later patterns than EHS (58 vs. 29 total overlaps). In EHI, overlaps were highest in SD (27) and DU (16), with smaller overlaps in TU (8) and DD (7). In EHS, overlap was concentrated in DD (15) with limited overlap in DU (5), SD (4), and TU (5). Directionally, EHS overlaps were predominantly hypomethylated, with EHS DU and EHS SD composed entirely of hypomethylated genes, whereas EHI overlap sets showed a mixed composition. Recurrent loci across patterns/conditions suggest shared epigenetic differences represented across multiple trajectories.

**Table 3:** Persistence of T0 differential expression into longitudinal response patterns between EHS and EHI. Orange = hyper-methylated (EHS vs. EHI at T0) and Blue = hypo-methylated (EHS vs. EHI at T0)

File	Count	Overlaps
EHI DD	7	ADARB2, CDYL2, IFT140, JARID2, KCNN3, MUC12, RPTOR
EHI DU	16	ADARB2, BACH2, BCL2, CCDC26, GAS2, GNA12, GRID1, IQGAP2, JARID2, LOC101928767, MED13L, PEG3, PIK3CD, PTPRN2, SH3TC2, TNIP3
EHI SD	27	ADARB2, CA10, CCKBR, CDYL2, DCHS2, DSCAML1, EPHA10, GNA12, HLA-DQB2, IL1R1, IQGAP2, KIAA0319, KLHL29, KNDC1, LARGE, MYO5B, MYOM2, NCK2, PDZRN3, PIK3CD, PRDM15, PTPRN2, RERE, RPTOR, SARDH, SLC43A2, SYTL3
EHI TU	8	CYTH1, ERBB4, IFT140, IRF6, LOC399886, MLLT10, SEMA4B, SLX4IP
EHS DD	15	ADARB2, ADPRHL1, BAHCC1, CCDC12, CCKBR, DCHS2, DSCAML1, HLA-DQB2, IFT140, KANSL1, MYT1L, PTPRN2, SCN1A, SH2B2, SLFN12
EHS DU	5	BCL2, CCDC12, DENND2D, PACS1, SLFN12
EHS SD	4	BACH2, EGLN3, EXT1, JARID2
EHS TU	5	LMX1B, NCRNA00094, PTPRN2, RPTOR, SERPINE2

Note: multiple loci mapping to the same gene may follow different longitudinal patterns; thus, the same gene symbol can appear in multiple patterns

**Fig. 3:**
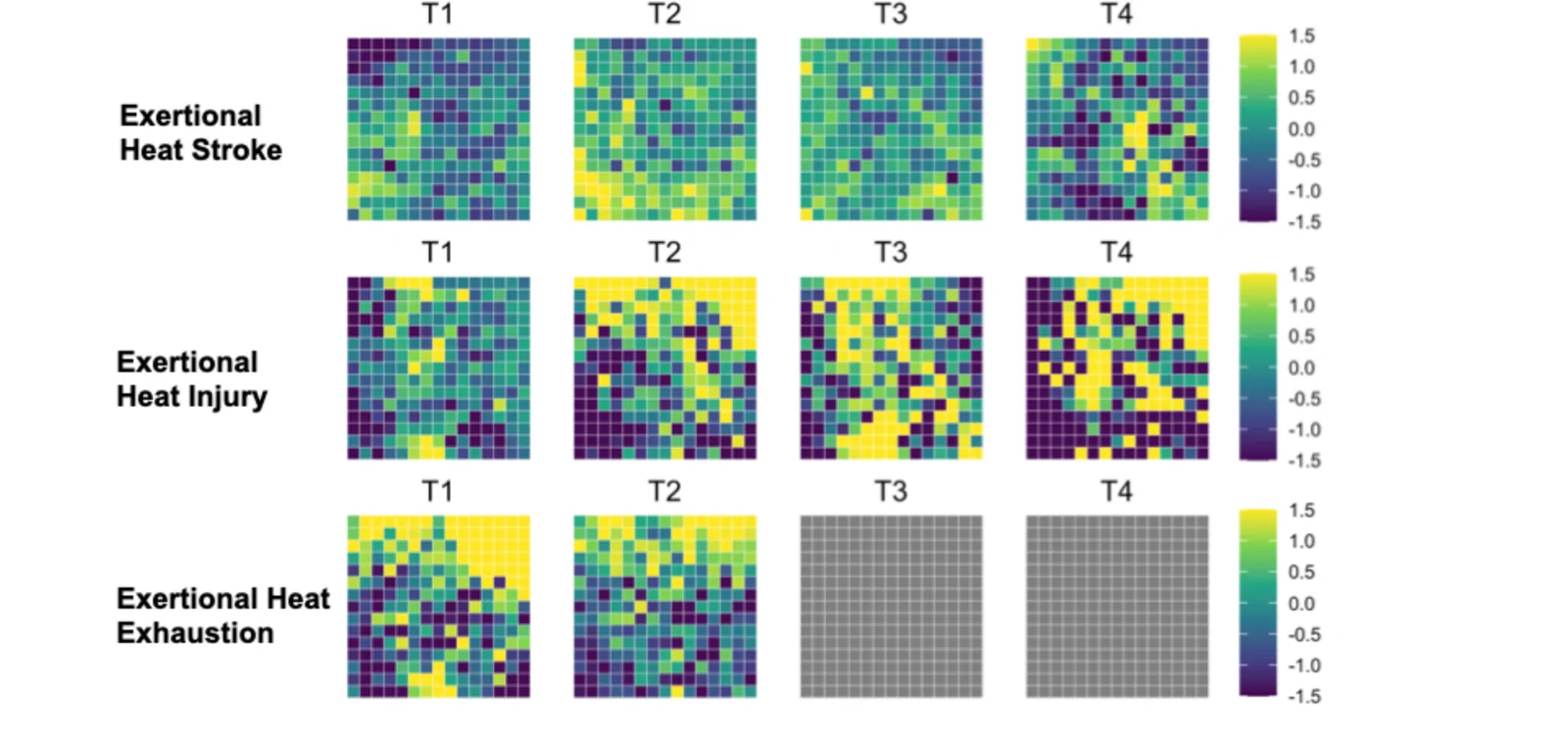
Self-Organizing Maps visualizing the global DNA methylation patterns for Exertional Heat Stroke, Heat Injury, and Heat Exhaustion across four time-bins (T1-T4). Each grid represents the average epigenetic state, with yellow indicating higher methylation and dark purple indicating lower methylation. The persistent changes in EHS and EHI contrast with the rapid resolution in EHE, where insufficient samples were available for later time points (gray grids)

For longitudinal analysis, individual sample collection days were grouped into four broader time bins. The SOMs provided a global visualization of the epigenetic evolution in each group over the four time-bins (Fig. 3). The time bins were chosen to represent distinct biological phases of the response to heat illness: the acute phase (T1: days 1–2), the sub-acute response (T2: days 3–4), early recovery (T3: days 5–8), and later recovery (T4: >8 days). The EHS and EHI groups displayed dramatic and persistent changes in their methylation profiles from T1 to T4. In contrast, the EHE group showed a much milder initial change and a more rapid return towards a baseline state, with insufficient samples for analysis at later time points (T3 and T4).

### Time-course clustering of temporal signatures

**Fig. 4:**
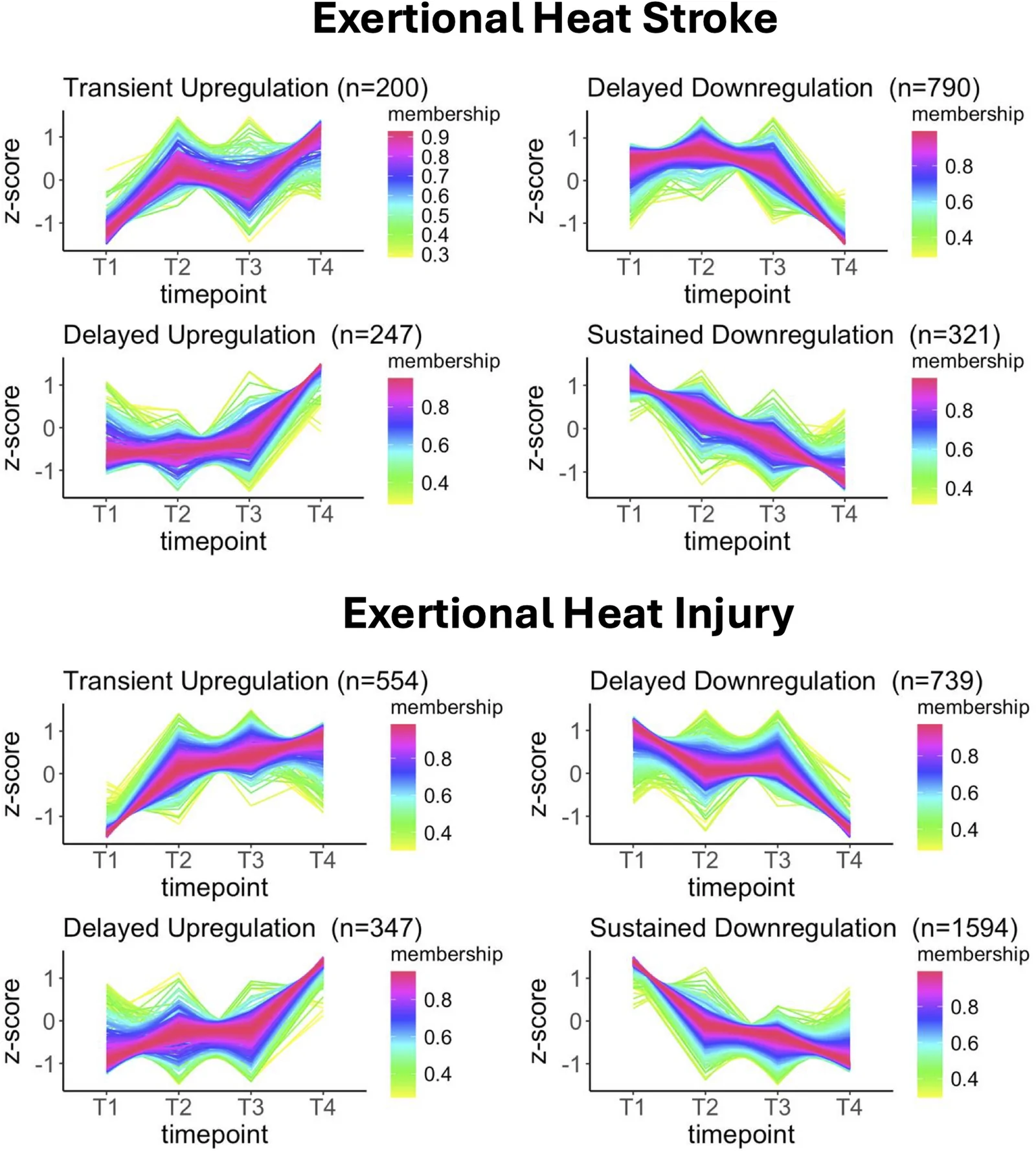
Temporal clustering of DNA methylation profiles for EHS and EHI. Each plot shows a distinct cluster of CpG probes with a similar methylation trajectory (z-score) across the four timepoints. The color of each line indicates the strength of its membership within that cluster

**Fig. 5:**
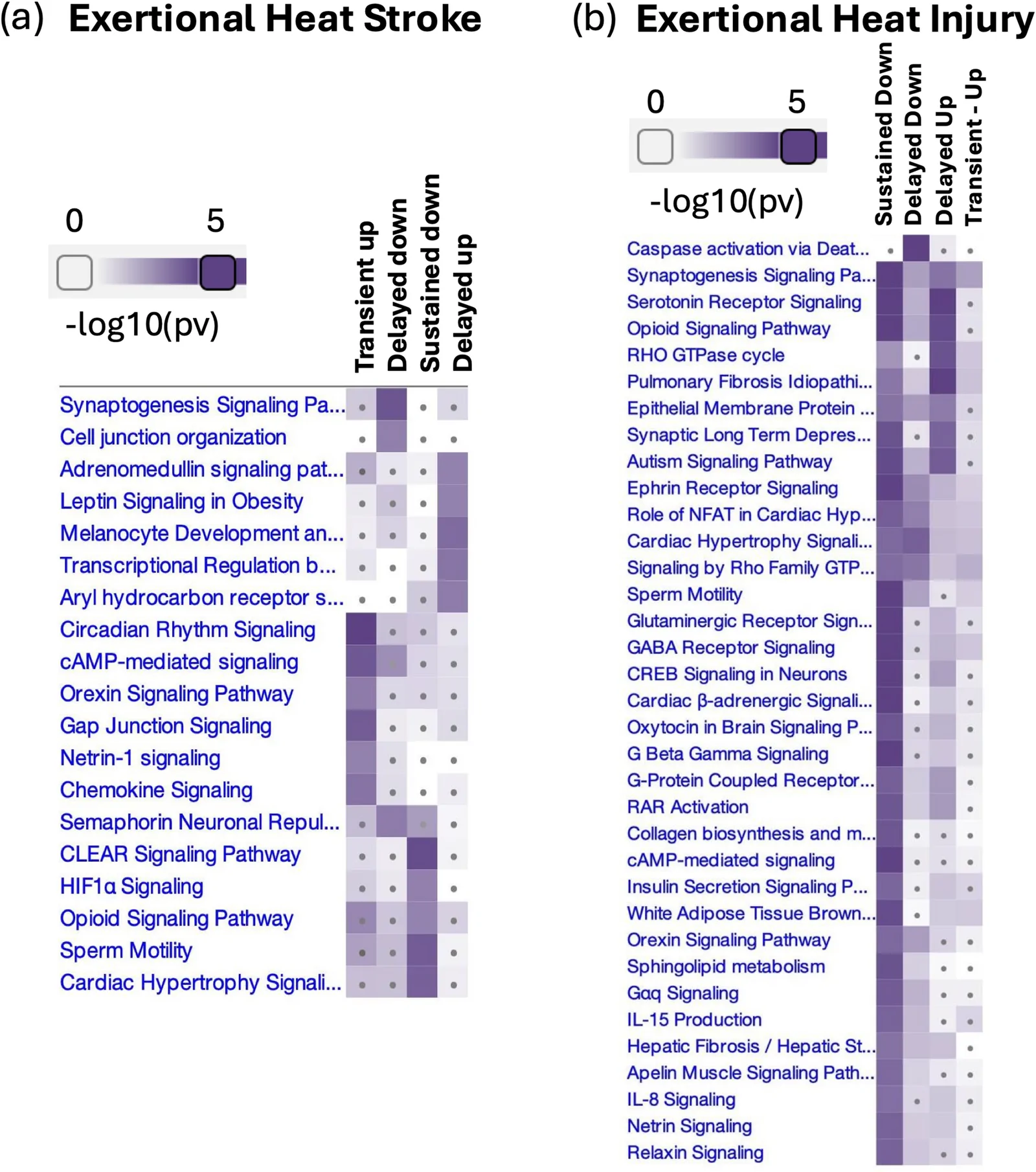
Hierarchical clustering of enriched canonical pathways for the temporal clusters identified in **a** Exertional Heat Stroke and **b** Exertional Heat Injury. The color intensity of the heatmap represents the statistical significance as -log10(p-value), where a darker color indicates higher significance

To complement the SOM analysis, we also identified distinct clusters of CpG sites with co-regulated temporal patterns for both EHS and EHI using the TCseq package (Fig. 4). In EHS, four primary clusters were observed: transient upregulation (*n* = 200), delayed upregulation (*n* = 247), delayed downregulation (*n* = 790), and sustained downregulation (*n* = 321). Similarly, EHI displayed four clusters, including sustained downregulation (*n* = 1,594), delayed downregulation (*n* = 739), delayed upregulation (*n* = 347), and transient upregulation (*n* = 554). To determine the potential biological significance of the temporal methylation clusters, we performed canonical pathway enrichment analysis for each TCseq-defined cluster and visualized the results by hierarchical clustering (Fig. 5). Because methylation was measured in bulk peripheral whole blood, these pathway findings are interpreted as whole-blood/leukocyte regulatory signatures rather than direct methylation changes in non-blood tissues. In EHS, temporal clusters were enriched for pathways related to inflammatory signaling, inflammasome/pyroptotic activation, oxidative stress, proteostasis, vascular and hemostatic regulation, hypoxia response, and immune-cell signaling. These findings suggest that EHS is associated with persistent immune activation and stress-response dysregulation during recovery. In contrast, EHI clusters showed pathway enrichment patterns consistent with chromatin remodeling, regulated inflammatory signaling, cytoskeletal organization, glucose transport and metabolic support, vascular tone, and ion/osmotic homeostasis. These patterns suggest a more adaptive recovery program in EHI compared with the more sustained inflammatory and metabolic disruption observed in EHS. Overall, the pathway analysis in indicates that EHI and EHS diverge over time, with EHI showing a more regulated recovery-associated methylation program and EHS showing persistent dysregulation of immune, oxidative, proteostasis, vascular, and metabolic pathways.

## Discussion

In the late 1970s, Pandolf and colleagues proposed the idea of a physiological memory in heat acclimation, suggesting genomic flexibility in response to heat stress [26]. Indeed, re-acclimation after a period of de-acclimation occurs more rapidly than initial acclimation, which could be due to epigenetic changes [18, 27, 28]. While heat acclimation decreases physiological strain in response to an acute heat exposure, extreme heat exposure can leave lasting epigenetic changes that weaken immune function and increase vulnerability to subsequent heat stress, which may have implications for risk of future heat illness [29]. For example, in rodents, the methylation response to EHS is associated with severe metabolic imbalances [10, 19–21]. Our study extends the investigation of heat stress-induced epigenetic changes to humans, focusing on how whole blood methylation patterns following EHE, EHI, and EHS.

### Metabolic regulation and phase-specific epigenetic responses to heat stress

At the initial time point (T0), only seven DMPs were shared across all groups. Pathway enrichment analysis indicated that these loci were primarily associated with the glutamatergic receptor signaling pathway, falling within broader categories of neurotransmitter and synaptic regulation. Among these, corticotropin-releasing hormone (CRH) was identified, and is notable for its role in activating the hypothalamic–pituitary–adrenal (HPA) axis, stimulating adrenocorticotropic hormone and cortisol release during heat stress, which aligns with prior findings [30, 31]. Our results suggest progressive hypermethylation of CRH promoter regions as heat stress severity increases. Since promoter methylation generally suppresses transcription, this pattern may indicate negative feedback on HPA-axis activation following initial CRH upregulation during acute stress. This could represent a regulatory shift from acute endocrine activation (in the milder EHE) toward neuroendocrine suppression (in the more severe EHS) to prevent cortisol overexposure.

The presence of hexokinase 2 (HK2), a key glycolytic enzyme, implicates metabolic adaptation [32]. Specifically, promoter hypomethylation in EHS relative to EHE suggests potentially higher HK2 transcriptional activity in severe heat illness, possibly as an adaptive metabolic response to increased ATP demand. Hypomethylation in EHI relative to EHE could reflect an intermediate stress response with impaired metabolic compensation. The presence of holocarboxylase synthetase (HLCS), an enzyme that biotinylates carboxylases involved in energy and lipid metabolism, further suggests an epigenetic–metabolic coupling mechanism during heat stress [33]. Hypomethylation in EHS and EHI compared to EHE indicates a potential upregulation of HLCS activity, supporting enhanced metabolic flux and chromatin stabilization during acute stress, whereas hypermethylation in EHS relative to EHI may reflect a transition toward metabolic downregulation and recovery once immediate energetic demands subside. Together, these shared methylation patterns across CRH, HK2, and HLCS indicate a coordinated neuroendocrine and metabolic response to thermal stress, where acute activation of stress and energy pathways during early heat exposure transitions toward progressive epigenetic repression as severity increases.

In contrast to the few loci shared across all conditions, several differentially methylated genes appeared exclusively in one or two groups, suggesting severity-specific regulatory mechanisms. For example, Transient Receptor Potential Cation Channel Subfamily M Member 2 (TRPM2) showed hypermethylation in EHS relative to EHE but hypomethylation in EHI relative to EHE, which is a ROS-activated cation channel involved in converting oxidative and heat stimuli into neuronal and thermoregulatory responses [34].Similarly, protein kinase C α (PRKCA) displayed opposite methylation directions across groups, showing hypermethylation in EHS relative to EHE but hypomethylation in EHI relative to EHE, suggesting biphasic regulation. Given PRKCA’s established role in vascular tone, cardiac contractility, and HSP70/72 induction, these methylation shifts may reflect initial activation of protective stress pathways during moderate heat injury followed by epigenetic suppression during severe heat illness to limit excessive calcium signaling and apoptotic signaling [35–37].

Antioxidant defense was also implicated by changes in Superoxide Dismutase (SOD) 3 methylation, which showed promoter hypermethylation in EHS relative to EHE but hypomethylation in EHI relative to EHE, suggesting that extracellular ROS-scavenging capacity may be initially upregulated during moderate heat injury but later suppressed in severe stress as oxidative burden exceeds compensatory mechanisms. This data is consistent with prior evidence that heat stress elevates ROS and SOD activity as part of the protective response [38, 39]. Nucleocytoplasmic transport also appears affected, as NUP62 exhibited consistent hypomethylation across EHS and EHI relative to EHE, indicating potential upregulation of nuclear pore function to facilitate HSP70 translocation and transcriptional recovery during hyperthermia-induced proteotoxic stress [40].

Finally, circulatory strain is supported by methylation changes in Hemoglobin subunit beta (HBB), which had promoter hypomethylation in both EHI and EHS compared to EHE, potentially indicating increased transcriptional potential of hemoglobin genes under progressively more severe thermal stress. This pattern aligns with evidence that HBB expression rises during hyperthermia as part of a compensatory response to enhance oxygen transport and tissue perfusion, reflecting systemic attempts to preserve oxygen delivery during heat-induced circulatory compromise [41].

### Early activation of immune and stress signaling

The transient response differs between EHS and EHI. In EHS, interferon-inducible genes are upregulated, consistent with activation of innate immune signaling during heat stress [42]. At the same time, cytotoxic effectors Perforin 1 (PRF1) and Granzyme B (GZMB) are also upregulated, potentially aimed at clearing heat-damaged cells [43]. In our data, stress-related signals were amplified by kinase hubs including Mitogen-Activated Protein Kinase 1 (MAPK1), FYN Poroto-oncogene (FYN), and Calcium/Calmodulin-Dependent Protein Kinase ID (CAMK1D), which coordinate kinase and calcium-dependent responses [44]. Finally, transcription factors RUNX Family Transcription Factor 3 (RUNX3) and Basic Helix-Loop-Helix Family Member E40 (BHLHE40) contribute through T-cell differentiation and cytokine production, thereby shaping both the character of the T-cell response and the magnitude of inflammatory signaling [45, 46]. By contrast, EHI seems to emphasize repair and regulation via gene changes: Brain derived neurotrophic factor (BDNF) supports neuronal survival and plasticity [47], FKBP Prolyl Isomerase 5 (FKBP5) dampens glucocorticoid receptor signaling to regulate stress hormone sensitivity [48], and Major Histocompatibility Complex, Class II, DP Beta 1 (HLA-DPB1) promotes antigen presentation for adaptive immune responses [49]. In addition, epigenetic modifiers such as HDAC4 and Lysine Demethylase 2B (KDM2B) influence which repair and immune genes are accessible [11]. Overall, EHS is characterized by broad immune and signaling activation, while EHI appears to reflect the initiation of adaptive programs linked to neuronal protection, hormone signaling, and antigen presentation.

### Progressive suppression of neuronal, vascular, and immune functions

The delayed downregulation response also differs between EHS and EHI. In EHS, suppression of neuronal and vascular pathways was prominent. Genes such as Microtubule-Associated Protein Tau (MAPT) and Calcium/Calmodulin-Dependent Protein Kinase II Alpha (CAMK2A) were hypermethylated, indicating reduced synaptic signaling and plasticity that may contribute to acute neurological dysfunction during heat stroke [50]. At the same time, vascular and hemostatic regulators, including Glycoprotein VI Platelet (GP6) and von Willebrand Factor (VWF), were suppressed, consistent with impaired platelet adhesion and endothelial repair, processes known to be disrupted in severe hyperthermia [51, 52]. Immune components such as MEFV Innate Immunity Regulator, Pyrin (MEFV) and HLA-DQB2 were also delayed [53], suggesting dampened inflammasome activity and antigen presentation, while reductions in Unc-51 Like Autophagy Activating Kinase 1 (ULK1) and Proteinase 3 (PRTN3) may weaken autophagy and proteolytic clearance of damaged proteins [54]. By contrast, EHI showed hypermethylation of apoptotic, oxidative, and synaptic pathways. Suppression of Fas Cell Surface Death Receptor (FAS) and Fas Ligand (FASLG) suggests reduced apoptotic signaling through the death receptor pathway, a mechanism that has also been observed in blood-cell-derived diseases where epigenetic downregulation of FAS contributes to impaired immune regulation [55]. Furthermore, hypermethylation of Catalase (CAT) and Glutathione Peroxidase 4 (GPX4) implies limited antioxidant capacity under oxidative stress [56, 57]. Other genes, including SH3 and Multiple Ankyrin Repeat Domains 2 (SHANK2), Glutamate Metabotropic Receptor 5 (GRM5), and DLG Associated Protein 3 (DLGAP3), were also hypomethylated, pointing to impaired glutamatergic signaling and synaptic stability, consistent with the consequences of heat injury in animal models [58]. Additional suppression of Nuclear Factor of Activated T Cells 5 (NFAT5), which supports T-cell survival under osmotic stress [59], together with RUNX3, a regulator of T-cell differentiation [45], and AT-Rich Interaction Domain 5B (ARID5B), which modulates cytokine expression [60], suggests weakened transcriptional control of immune and stress-adaptive pathways.

### Adaptive remodeling of chromatin, metabolic, and inflammatory networks

In EHI, delayed upregulation appeared to emphasize regulatory and structural pathways that support recovery. Genes such as HDAC4, Jumonji and AT-Rich Interaction Domain Containing 2 (JARID2), and AT-Rich Interaction Domain 4B (ARID4B) point to chromatin remodeling and transcription during the later phases of adaptation [11]. Upregulation of IL1B and IL10 suggests continued but balanced immune signaling, potentially limiting excessive inflammation [61]. Plectin 1 (PLEC1) suggests reinforcement of the cytoskeleton to protect cell integrity during stress [62], while Protein Kinase cGMP-Dependent 1 (PRKG1) indicates regulation of vascular tone [63]. Upregulation of Rac Family Small GTPase 1 (RAC1) suggests enhanced trafficking of glucose transporters to the cell surface [64], while induction of Solute Carrier Family 2 Member 14 (SLC2A14), a high-affinity glucose transporter, may further support sustained glucose entry to preserve ATP production during mitochondrial recovery [65]. In parallel, Serine/Threonine Kinase 39 (STK39) encodes the kinase STE20/SPS1-Related Proline-Alanine-Rich Protein Kinase (SPAK), which activates ion transporters and the p38 MAPK stress pathway, thereby maintaining ionic balance and osmotic stability during stress [66]. Together, these changes indicate that EHI sustains energy availability and homeostatic control during recovery from heat injury. In EHS, the delayed upregulation reflects persistent activation of innate immune sensors, proteostasis networks, and stress-linked transcriptional regulators. Genes such as Caspase Recruitment Domain Family Member 8 (CARD8) and Gasdermin D (GSDMD) remain elevated, consistent with prolonged inflammasome activity and pyroptotic signaling that drive systemic inflammation and cell death during severe hyperthermia [67, 68]. Epigenetic modifiers, including HDAC4 in EHI-associated methylation clusters and HDAC2 in EHS-associated methylation clusters, suggest chromatin remodeling and transcriptional reorganization during recovery from heat illness [69, 70]. CpGs annotated to HSP90AB1 suggest altered methylation of proteostasis-related pathways during early recovery from EHS and prior murine work has shown reduced heat shock transcript responses in immune cells following a secondary heat exposure 30 days after EHS, suggesting that early post-injury methylation patterns and longer-term transcriptional responses to re-exposure may represent distinct phases of the heat-stress response [71]. Persistent upregulation of TXNRD1 suggests cells remain under oxidative strain, as this enzyme is mobilized to counteract excess ROS and maintain redox balance [72]. At the same time, elevated TIMP Metallopeptidase Inhibitor 2 (TIMP2) indicates potential extracellular matrix remodeling; while protective in stabilizing tissue structure, prolonged TIMP2 activity may impair normal repair and contribute to maladaptive vascular changes [73]. However, the functional consequences of these changes cannot be classified as adaptive or maladaptive based on methylation data alone and require broader validation. These findings therefore support the presence of severity-associated differences in chromatin-regulatory methylation patterns rather than a definitive direction of remodeling.

### Divergent long-term epigenetic programs: adaptation vs. systemic dysfunction

In EHI, sustained downregulation highlights a shift toward cellular stress management. Suppression of GCN2 suggests activation of the integrated stress response, a program that reduces protein synthesis to conserve resources under nutrient and oxygen limitation [74, 75]. Downregulation of GRM5, SHANK1/2, and Nueroligin 1 (NLGN1) points to weakened glutamatergic receptor signaling and synaptic scaffolding, consistent with impaired neuronal plasticity and the potential for lasting cognitive changes following heat injury [76, 77]. Immune regulators, including AKT1/2, PIK3R5, and LCK, are also reduced, suggesting dampened T-cell receptor signaling and a slow return to normal [78, 79]. Together, these changes suggest that EHI shifts cells into a resource-conserving state while limiting synaptic activity and immune responsiveness, consistent with a recovery trajectory rather than systemic collapse. By contrast, EHS sustained downregulation reflects the failure of metabolic, cardiovascular, and neuronal systems. Reduced expression of EGLN3, the primary oxygen sensor in the HIF-1α pathway, points to impaired hypoxia signaling [80]. Downregulation of cardiac regulators such as PPP3CA (calcineurin), HDAC4, and MEF2D indicates disruption of cardiac hypertrophy signaling and impaired adaptation to circulatory strain [81, 82]. Concurrent suppression of RAC2, PRKD1, and BLK implicates weakened immune and vascular signaling [83, 84]. Thus, while EHI represents a resource-conserving, stress-adaptive program, EHS reflects systemic dysfunction that may drive longer-term organ and cognitive challenges.

### Current and emerging targets for managing heat stroke and epigenetic recovery

Current best practices for treating heat stroke are centered on rapid cooling and supportive care. Target-enrichment analyses highlight candidate druggable nodes in calcium handling, oxidative stress, and inflammasome signaling. In this context, dantrolene—a ryanodine receptor antagonist FDA-approved for malignant hyperthermia—has received attention for its ability to reduce intracellular calcium release and muscle-derived heat production [85]. Case reports and small clinical trials suggest that dantrolene may modestly accelerate cooling in exertional heat stroke, and pretreatment studies in animal models show reductions in severity and organ injury [86]. However, larger controlled trials in humans, including classic heat stroke, have failed to demonstrate improvements in survival or long-term neurological outcomes. Importantly, the pathophysiology of malignant hyperthermia is quite distinct from that of EHS, so any overlap in potential treatment should be carefully evaluated [1, 87].

Muscle relaxants, benzodiazepines, and neuroleptic agents have been used clinically to inhibit shivering [87], an involuntary thermogenic process that can delay cooling and increases the potential for metabolic imbalances. These interventions align with our data showing dysregulation of calcium and kinase signaling pathways, suggesting that reducing involuntary muscle activity could directly limit heat production and downstream cellular injury. However, shivering only occurs in a subset of patients and is only relevant during the immediate post-collapse active cooling period. At the same time, other epigenetic modifiers are emerging as potential adjuncts. Histone deacetylase inhibitors (HDACi) such as vorinostat, romidepsin, and belinostat are currently approved for hematologic malignancies, and in a recent Caenorhabditis elegans model, low-dose vorinostat enhanced resistance to oxidative and heat stress, improving survival and healthspan [88]. While romidepsin and belinostat have not yet been evaluated in heat stress contexts, their capacity to modulate transcriptional programs linked to inflammation, proteostasis, and neuronal survival suggests potential therapeutic relevance. Other targets such as BDNF and FKBP5 implicate neuronal plasticity and glucocorticoid receptor regulation, respectively, pointing to opportunities for neuroprotective or stress-axis–modulating agents (e.g., TrkB agonists, SAFit2) to support long-term recovery. While these remain speculative and are not part of current clinical guidelines, their identification provides a roadmap for future studies.

### Limitations and conclusion

Our study has limitations. While we were fortunate to evaluate samples from an unprecedented number of patients over the spectrum of heat illness (EHE, EHI and EHS), we were not able to include a control group. Fortunately, heat illness is not common – thus, we would have had to follow thousands of individuals during training to have meaningful controls doing the same thing, at the same time, as the individuals who collapsed. This was not feasible within the logistical and resource constraints of the present work. Future studies should include healthy resting controls, exercise-matched controls, and heat-exposed controls without clinical heat illness to isolate methylation signatures specific to exertional heat illness severity. Another limitation is our whole-blood methylome analyses which aggregates signals across cell types and do not capture tissue-specific events; thus, deconvolution, multi-omic integration (ATAC-seq, RNA-seq, proteomics), and single-cell approaches are needed to resolve cell-type effects. We also did not measure histone acetylation or noncoding RNAs, which are likely contributors to heat-stress epigenetics. The present sampling window primarily reflects acute and subacute recovery rather than long-term epigenetic remodeling. Although T4 captured samples collected beyond 8 days post-diagnosis, this timeframe is shorter than several murine EHS studies showing methylation changes approximately 30 days after injury. Based on these studies, we speculate that some acute inflammatory and stress-response methylation signatures would resolve over 2–3 months, whereas a subset of loci may persist, particularly following EHS. Future human studies with longer follow-up sampling are needed to determine whether these methylation patterns represent transient methylation responses or epigenetic memory associated with recurrent heat illness risk or long-term organ complications. Nonetheless, convergence with murine data strengthens inference and frames testable hypotheses: (i) whether specific CpG signatures predict clinical recovery, recurrence risk, or organ complications; (ii) whether targeted countermeasures (e.g., rapid cooling, drugs) can change maladaptive methylation trajectories; and (iii) whether heat acclimation, fitness, sex, or prior EHI history shape the methylation response.

Taken together, our findings highlight that heat stress induces both conserved and divergent molecular programs across the spectrum of heat illness. Early responses converge on shared nodes of thermal stress, antioxidant defense, and metabolic support, but trajectories diverge thereafter: EHI engages adaptive programs that emphasize repair, recovery, and immune regulation, whereas EHS progresses toward proteotoxic stress, vascular dysfunction, and immune collapse. Epigenetic changes, particularly in DNA methylation, may provide a molecular memory that shapes susceptibility to future heat illness and distinguishes recovery from pathology. Ultimately, this work advances the understanding of how heat stress influences epigenetics and points to new opportunities for biomarker development, drug discovery, and targeted interventions.

## Supplementary Information

Below is the link to the electronic supplementary material.


Supplementary Material 1.


## Data Availability

The data from the current study are only available with an approved data sharing agreement requested through the corresponding author due to military institute regulations.
