# Supplementary material for: Dynamic responses in the human methylome to exertional heat exhaustion, heat injury, and heat stroke
Source: Hum Genomics. 2026 Jun 19;20:131. doi: 10.1186/s40246-026-01008-8 (PMC13527912; doi:10.1186/s40246-026-01008-8)
Supplement: Supplementary file 1 [file 40246_2026_1008_MOESM1_ESM.docx]

**Figure S1**. Top 20 DMPs distinguishing Exertional Heat Stroke (EHS) from Exertional Heat Exhaustion (EHE).

**Figure S2**. Top 20 DMPs distinguishing Exertional Heat Injury (EHI) from Exertional Heat Exhaustion (EHE).

**Figure S3**. Top 20 DMPs distinguishing Exertional Heat Stroke (EHS) from Exertional Heat Injury (EHI).

Table S1. DNA Methylation-Estimated Blood Profiling at Baseline (Time = 1)

| **Genomic Feature** | **EHS vs. EHE (N = 2,258)** | **EHI vs. EHE (N = 628)** | **EHS vs. EHI (N = 424)** |
| --- | --- | --- | --- |
| Body | 836 (37.0%) | 242 (38.5%) | 144 (34.0%) |
| IGR | 643 (28.5%) | 217 (34.6%) | 151 (35.6%) |
| TSS1500 | 305 (13.5%) | 72 (11.5%) | 55 (13.0%) |
| 5'UTR | 218 (9.7%) | 37 (5.9%) | 38 (9.0%) |
| TSS200 | 148 (6.6%) | 37 (5.9%) | 16 (3.8%) |
| 1stExon | 52 (2.3%) | 9 (1.4%) | 11 (2.6%) |
| 3'UTR | 50 (2.2%) | 11 (1.8%) | 9 (2.1%) |
| ExonBnd | 6 (0.3%) | 3 (0.5%) | 0 (0.0%) |

Table S2. Longitudinal Cell Profiling Across Time Bins Cell Profiling.

| Cell Type | T1 (N = 118) | T2 (N = 46) | T3 (N = 59) | T4 (N = 46) |
| --- | --- | --- | --- | --- |
| Neutrophils (Neu) | 66.32% ± 11.77% | 59.61% ± 8.23% | 59.30% ± 10.09% | 59.06% ± 8.78% |
| Monocytes (Mono) | 9.43% ± 2.69% | 10.31% ± 2.48% | 9.97% ± 2.68% | 10.38% ± 2.25% |
| CD8 + T Cells (CD8T) | 9.37% ± 4.70% | 11.58% ± 3.80% | 12.29% ± 4.13% | 11.30% ± 4.24% |
| CD4 + T Cells (CD4T) | 7.48% ± 4.70% | 10.96% ± 3.87% | 10.52% ± 3.94% | 9.71% ± 3.81% |
| B Cells (Bcell) | 6.05% ± 3.22% | 7.73% ± 3.28% | 8.55% ± 3.32% | 8.79% ± 3.58% |
| NK Cells (NK) | 4.15% ± 4.40% | 2.84% ± 2.68% | 2.81% ± 2.48% | 4.32% ± 3.20% |

Table S3. Exertional Heat Stroke Longitudinal Cohort Cell Profiling (N = 186 samples)

| Cell Type | T1 (N = 53) | T2 (N = 37) | T3 (N = 53) | T4 (N = 43) |
| --- | --- | --- | --- | --- |
| Neutrophils (Neu) | 66.46% ± 13.20% | 59.84% ± 8.90% | 59.46% ± 10.36% | 60.09% ± 8.10% |
| Monocytes (Mono) | 8.68% ± 2.62% | 10.47% ± 2.50% | 9.80% ± 2.66% | 10.24% ± 2.26% |
| CD8 + T Cells (CD8T) | 9.47% ± 5.74% | 11.40% ± 3.86% | 12.35% ± 4.35% | 10.78% ± 3.87% |
| CD4 + T Cells (CD4T) | 6.71% ± 5.07% | 11.21% ± 4.12% | 10.44% ± 3.88% | 9.58% ± 3.90% |
| B Cells (Bcell) | 6.40% ± 3.46% | 7.82% ± 3.45% | 8.83% ± 3.37% | 8.68% ± 3.66% |

Table S4. Exertional Heat Injury Longitudinal Cohort Cell Profiling (N = 38 samples)

| Cell Type | T1 (N = 23) | T2 (N = 6) | T3 (N = 6) | T4 (N = 3) |
| --- | --- | --- | --- | --- |
| Neutrophils (Neu) | 69.82% ± 10.12% | 58.91% ± 5.52% | 57.87% ± 7.86% | 44.36% ± 3.10% |
| Monocytes (Mono) | 9.62% ± 2.42% | 10.29% ± 2.72% | 11.51% ± 2.55% | 12.36% ± 0.24% |
| CD8 + T Cells (CD8T) | 8.48% ± 3.36% | 11.96% ± 4.36% | 11.72% ± 0.87% | 18.74% ± 0.69% |
| CD4 + T Cells (CD4T) | 6.42% ± 4.11% | 9.96% ± 3.14% | 11.28% ± 4.74% | 11.57% ± 0.90% |
| B Cells (Bcell) | 4.99% ± 2.15% | 7.19% ± 3.17% | 6.10% ± 1.30% | 10.41% ± 1.28% |
| NK Cells (NK) | 3.56% ± 2.23% | 4.90% ± 2.14% | 4.71% ± 2.28% | 7.26% ± 1.38% |

Table S5. Genomic Feature Descriptive Analysis.

| **Genomic Feature** | **EHS vs. EHE (N = 2,258)** | **EHI vs. EHE (N = 628)** | **EHS vs. EHI (N = 424)** |
| --- | --- | --- | --- |
| Body | 836 (37.0%) | 242 (38.5%) | 144 (34.0%) |
| IGR | 643 (28.5%) | 217 (34.6%) | 151 (35.6%) |
| TSS1500 | 305 (13.5%) | 72 (11.5%) | 55 (13.0%) |
| 5'UTR | 218 (9.7%) | 37 (5.9%) | 38 (9.0%) |
| TSS200 | 148 (6.6%) | 37 (5.9%) | 16 (3.8%) |
| 1stExon | 52 (2.3%) | 9 (1.4%) | 11 (2.6%) |
| 3'UTR | 50 (2.2%) | 11 (1.8%) | 9 (2.1%) |
| ExonBnd | 6 (0.3%) | 3 (0.5%) | 0 (0.0%) |
